# Intra-articular Injection of Chitosan-Based Supramolecular Hydrogel for Osteoarthritis Treatment

**DOI:** 10.1007/s13770-020-00322-z

**Published:** 2021-01-28

**Authors:** Donggang Mou, Qunying Yu, Jimei Zhang, Jianping Zhou, Xinmin Li, Weiyi Zhuang, Xuming Yang

**Affiliations:** 1 Department of Orthopedics, Yan’an Hospital Affiliated to Kunming Medical University, Kunming, 650035 People’s Republic of China; 2 Department of Maternity, The Second Affiliated Hospital of Kunming Medical University, Kunming, 650051 People’s Republic of China; 3 Department of Gastroenterology, Chenggong Hospital, Yan’an Hospital Affiliated to Kunming Medical University, Kunming, 650035 People’s Republic of China; 4 Department of Orthopedics, Chenggong Hospital, Yan’an Hospital Affiliated to Kunming Medical University, Kunming, 650035 People’s Republic of China; 5 Department of Cardiology, Chenggong Hospital, Yan’an Hospital Affiliated to Kunming Medical University, Kunming, 650035 People’s Republic of China

**Keywords:** Chitosan, Hyaluronic acid, Hydrogel, Osteoarthritis, Intra-articular injection

## Abstract

**Background:**

Pain and cartilage destruction caused by osteoarthritis (OA) is a major challenge in clinical treatment. Traditional intra-articular injection of hyaluronic acid (HA) can relieve the disease, but limited by the difficulty of long-term maintenance of efficacy.

**Methods:**

In this study, an injectable and self-healing hydrogel was synthesized by in situ crosslinking of *N*-carboxyethyl chitosan (*N*-chitosan), adipic acid dihydrazide (ADH), and hyaluronic acid–aldehyde (HA-ALD).

**Results:**

This supramolecular hydrogel sustains good biocompatibility for chondrocytes. Intra-articular injection of this novel hydrogel can significantly alleviate the local inflammation microenvironment in knee joints, through inhibiting the inflammatory cytokines (such as TNF-α, IL-1β, IL-6 and IL-17) in the synovial fluid and cartilage at 2- and even 12-weeks post-injection. Histological and behavioral test indicated that hydrogel injection protected cartilage destruction and relieved pain in OA rats, in comparison to HA injection.

**Conclusion:**

This kind of novel hydrogel, which is superior to the traditional HA injection, reveals a great potential for the treatment of OA.

## Introduction

Osteoarthritis (OA) is recognized as a degenerative joint disorder, and it is the most common kind of the arthritis, characterizing by affecting stability of cartilage, integrity of the synovial membrane, and bone structure progressively, which lead to chronic pain and disability [1]. The cartilage undertakes gradual impair and calcified erosion, while the extracellular matrix constituents are compromised which dramatically influence the tensile strength and resistance of cartilage. Furthermore, proinflammatory cytokines relieved by chondrocytes deteriorate the synovial tissue degradation, leading to a chronic inflammation and joint dysfunction [2].

There is an emerging treatment for OA, which is drug administration by intra-articular (IA) injection [3]. The strategy of drug delivery minimizes the systemical influence, and optimizes local advantages. While, traditional oral drugs administrated by IA injection are restricted by the short-term release. Recently, injectable materials, such as hydrogel microspheres or hydrogels have been extensively explored for their applications as IA injection for the cure of OA, which are attributed to the properties of their pimping toxicity, high loading efficiency and extended drug relieving time [4]. In healthy joints, the synovial membrane plays an essential role in joint lubrication by secreting hyaluronic acid (HA) and lubricin into the synovial fluid. While, in joints of OA, the concentration and condition of polymerization of HA influence the viscosity and composition of the synovial fluid [3]. IA injection of HA is recognized as a cost-effective strategy as viscosupplementation (VS) to treat OA symptoms and to conserve the joint function [4]. HA is a biopolymer naturally presenting in the synovial fluid, and several kinds of HA hydrogels are currently available applied for intra-articular injection of osteoarthritis, confirmed with a certain effect [5]. However, the half-life of HA is short as 1–2 days in the tissue, causing the drugs incorporated HA hydrogels with a slightly shorter retention time in joints, thus limiting its applications as VS therapy [4, 6]. Additionally, complications are prone to be caused for the frequent IA administration, such as infection, tissue injury, and it is difficult to sustain a long-term stable efficacy [7]. While, cross-linked HA hydrogels, which is synthesized by targeting the carboxyl and hydroxyl groups of the glucuronic acid or developing cysteine derived HA, can improve biological properties and prolong sustaining time effectively [8].

Chitosan and its derivatives have been widely applied in the fields of tissue engineering as well as medicine. Chitosan, which is comprised of copolymers of glucosamine and *N*-acetylglucosamine units linked by β-1,4-glycosidic linkages, is a potential and emerging candidate for biomaterial that can be applied for VS [9, 10]. In fact, chitosan is considered as an ideal material for hydrogels due to its biocompatibility, progressive degradability, non-toxic, biological activity, anti-inflammatory effects and antibacterial activity. Another attractive characteristic of chitosan is the presence of amino, and hydroxyl groups can be easily reacted and chemically modified, thus allowing a high chemical versatility [11, 12]. Several previous studies have shown that chitosan has a chondroprotective function and could enhance chondrocyte proliferation, increase the expression level of cartilage matrix components and inhibit inflammatory and catabolic mediators after IA injection in OA models [12, 13]. Moreover, chitosan is prone to inhibiting cartilage degradation and synovial membrane inflammation [14].

**Scheme 1:**
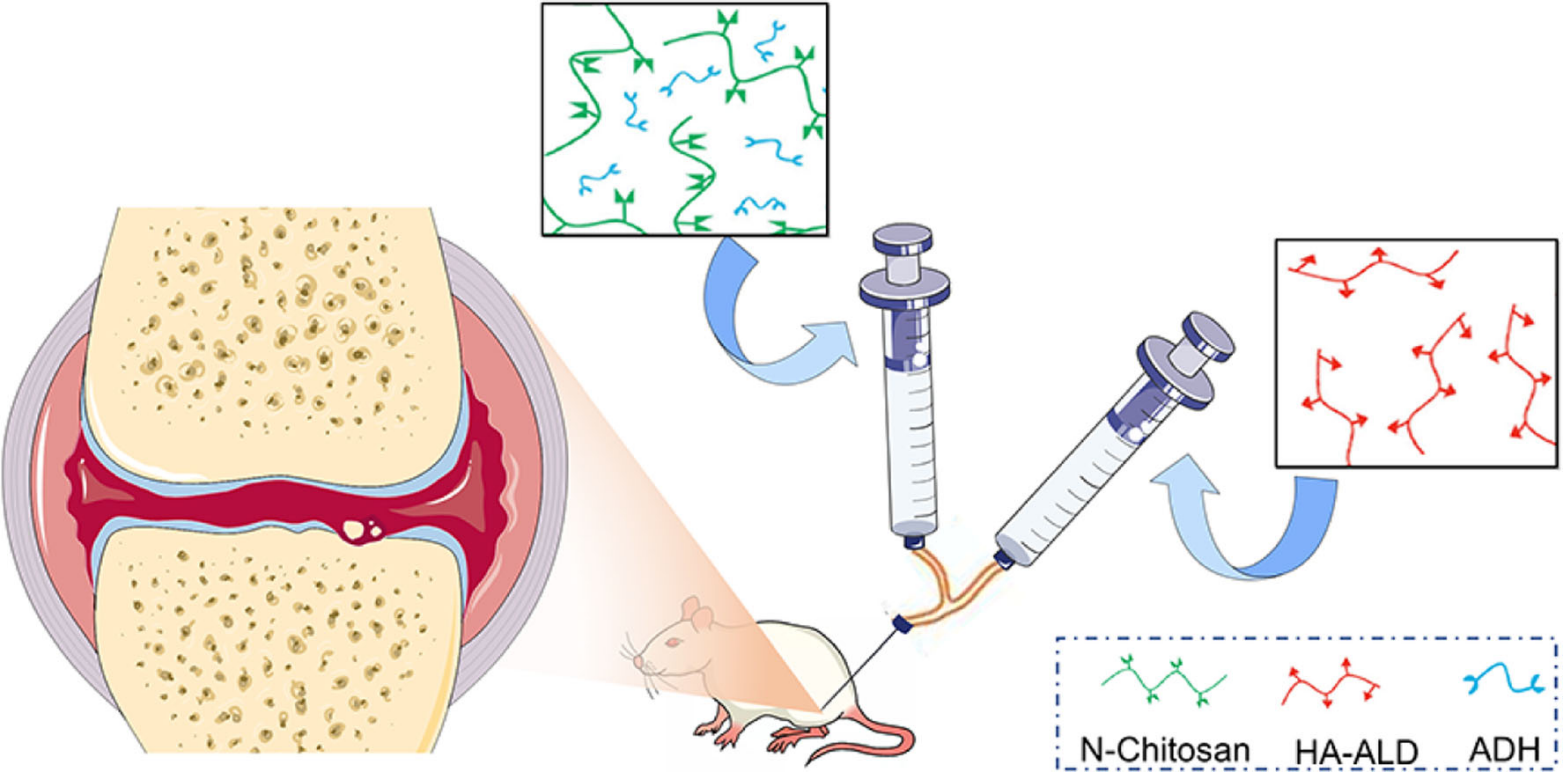
Chitosan-based hydrogel is prepared by in situ crosslinking of *N*-chitosan, ADH, and HA- ALD, and injected into the articular cavity of OA rats by a dual channel syringe to evaluate the therapeutic effects

In order to prolong retention time of hydrogels in joints and improve the outcome of OA, we prepared a novel supramolecular by in situ crosslinking of *N*-carboxyethyl chitosan (*N*-chitosan), adipic acid dihydrazide (ADH), and hyaluronic acid–aldehyde (HA-ALD). Our previous studies revealed that this supramolecular hydrogel contained good biocompatibility *in vitro* and *in vivo*, and possessed a long retention time and degraded slowly for up to 28 days *in vivo*, achieved good biological effects in the bone defects under rheumatoid arthritis and osteoporosis states [15, 16]. We speculated that injection of this chitosan-based hydrogel into the knee joints of OA rats could inhibit the inflammatory microenvironment, thus ameliorating cartilage damage and relieving pain through IA injection (Scheme 1). To the best of our knowledge, it is the first study to investigate the combination of synergistic action of HA-ALD associated with *N*-chitosan for IA injection in OA.

## Materials and methods

### Materials

Chitosan (degree of deacetylation ~ 95%, viscosity 100–200 mPa.s) was obtained from Aladdin (Shanghai, China). Sodium monoiodoacetate (MIA) and sodium periodate (NaIO4) were supplied by Sigma-Aldrich (St. Louis, MO, USA). Enzyme-linked immunosorbent assay (ELISA) kit of tumor necrosis factor (TNF)-α, interleukin (IL)-1β, IL-6, and IL-17 were purchased from Sigma-Aldrich. Hyaluronic acid (100–200 k) and adipic acid dihydrazide (ADH) were produced by Yuanye biology (Shanghai, China). Rat chondrocytes (ATCC-0038) was got from American Type Culture Collection (ATCC, Gaithersburg, MD, USA). Dulbecco’s Modified Eagle’s Medium (DMEM, low glucose), streptomycin–penicillin and fetal bovine serum (FBS) were supplied by Gibco^®^ Life Technologies (Carlsbad, CA, USA). Cell Counting Kit-8 (CCK-8) and Calcein-AM/Propidium Iodide (PI) were purchased from Beyotime Biotechnology (Shanghai, China). Hematoxylin–Eosin and Safranin O stains were got from Thermo Fisher Scientific Co., Ltd (Shanghai, China). Triton X-100 was supplied by Solarbio Science & Technology Co., Ltd. (Beijing, China). The antibodies enrolled in immunofluorescence were obtained from Abcam (Cambridge, UK).

### Preparation of chitosan-based hydrogel

The multifunctional hydrogel was manufactured as reported previously [16]. In concise, hydrogel was synthesized by in situ crosslinking of *N*-chitosan, ADH, and HA- ALD; 7.5% *N*-chitosan (w/v) and 7.5% ADH (w/v) were dissolved in deionized water. Then, solutions of 5% HA-ALD (w/v) were added to the above mixture. To form the hydrogel by imine and acylhydrazone bonds effects, the solutions crosslink for about 20 s. The self-healing properties of the hydrogel are tested by the rheological recovery test.

### Biocompatibility and degradation study

In order to study the biocompatibility, a thin layer of prepared hydrogel was placed on the bottom of the 48-well plates (hydrogel group) and the chondrocytes seeded in the plate directly as the control group (Con group). Briefly, chondrocytes were seeded at a density of 5 × 10^5^ cells/well and then incubated under the condition of 37 °C and 5% CO_2_ atmosphere.

After incubation for 1, 4, and 7 days, CCK-8 reagent was added into the samples and then incubated at 37 °C for 2 h. Finally, 100 μL solution of each specimen was transferred into the 96-well plate and measured at 450 nm by a microplate reader (Multiskan EX, Thermo Fisher Scientific, Shanghai, China). To evaluate the cell viability in the hydrogel, Live/Dead assay was performed at 4 days after chondrocytes seeding, the samples were cultured with 1 mM calcein-AM for 1 h and then stained with 1 ug/mL propidium iodide (PI) for 5 min at 37 °C. Next, the images were captured by fluorescence microscopy (Olympus IX71, Tokyo, Japan). *in vivo* degradation were carried out by subcutaneous injection of hydrogel in rats. In brief, 1 mL hydrogel was subcutaneously injected into the backs of Sprague–Dawley (SD) rats. The rats were sacrificed at the given time points, and the skin was harvested and fixed in 4% (W/V) paraformaldehyde solution. Hematoxylin and eosin (H&E) stained was conducted for *in vivo* biocompatibility analysis. All optical images were captured by the digital camera (Canon EOS 550D, Japan).

### Establishment of osteoarthritis and experimental design

All animal operating procedures are according to the guidelines for Institutional Animal Care and Use Committee of Kunming Medical University and approved by the Animal Ethics Committee of Yan’an Hospital Affiliated to Kunming Medical University (Approval No. 94R20B122).

Unilateral osteoarthritis was established under light anesthesia condition by single intra-articularly injecting monoiodoacetate (MIA, 3 mg/50 μl, soluted in 0.9% saline) in the right knee of the rats. Knee massage was conducted after injection to promise homogeneity, and OA model was developed 3 weeks post MIA injection [3]. Rats were randomly seperated into three groups (each group n = 15), namely, Group I: IA injection of saline (Untreated group); Group II: IA injection of HA (HA group); and Group III: IA injection of hydrogel (hydrogel group). Three weeks after MIA induction, treatment started and IA injection of different therapeutic agents (0.4 ml) were administered in the right knee once a week for three times. Behavioral tests were tested at 2 and 12 weeks of IA treatment, and the animals were euthanized for further examination.

### Inflammatory cytokines concentration detection in synovial fluid

Two and 12 weeks after IA injection of therapeutic agents, synovial fluid were extracted from articular cavity of these OA rats. Briefly, after these OA rats were sacrificed, the right knee joints were exposed. And then syringes are used to draw the joint fluid from the joint cavity. Totally 0.1 ml joint fluid was collected from each joint sample and added to 0.9 ml normal saline for dilution, and store at − 20 °C for testing. The concentration of TNF-α, IL-1β, IL-6, and IL-17 in the synovial fluid were detected by ELISA kits following the instructions of the manufacturer.

### Histological and immunohistochemical evaluation

At the time point of 2 and 12 weeks after IA injection therapies, these OA rats were sacrificed, and the right knee joints were collected carefully and fixed in 4% paraformaldehyde for further tests. Bone tissue samples of knee joint were decalcified with 0.5 M EDTA solution for 1 month and embedded for paraffin-sectioning to obtain slices with a thickness of 5 μm. Referring to the standard protocols, samples were stained with H&E and Safranin O. Histological changes were also evaluated through the scoring system which was developed by Kikuchi et al. [17]. The positive area of Safranine O staining was quantitatively analyzed by Image Pro Plus 6.0 (IPP, NIH, MD, USA). Immunofluorescence staining was performed to detect the expression of TNF-α, IL-1β, IL-6, and IL-17 in the cartilage and analyzed by IPP.

### Behavioral studies of the pain relief

In order to study the pain relief of IA injection, weight bearing capacity (WBC) test and paw withdrawal threshold (PWT) test were carried out to detect the gait and pain threshold [18]. A 3D gait analysis system (Kinama Tracer, Japan) was used for quantifying the WBC of the hind limb of rabbits while freely walking. Briefly, the OA rats walked along the runway equipped with mechanical sensors. And the sensors could record the ground reaction force for each limb, which indicate the weight bearing of the corresponding limb. Under normal conditions, the weight of both limbs is equal. However, when inflammatory pain occurs in one joint, the rat will actively avoid weight bearing of the corresponding limb to reduce the pain. Therefore, the weight bearing detected by the 3D gait analysis system will be less than 50%. According to the ground reaction force, the weight-bearing indexs (WBI) were calculated by the following formula:

$${\text{WBI}}\, = \,{\text{Ipsilateral }}\,{\text{weight}}/({\text{Ipsilateral weight}}\, + \,{\text{Contralateral weight}})\, \times \, 100\%$$

In addition, to study the mechanical allodynia (hypersensitivity), PWT has been detected by a von Frey filament (Ugo Basile, Varese, Italy) as previous study [18]. Briefly, the withdrawal threshold was gauged by the force exerted by von Frey filament ranging from 0 to 40 grams with a 0.2 g accuracy. An acicular stimulation was delivered to the mid-planta pedis of right hind limbs through the meshy at the bottom of the cage and the withdrawal threshold was automatically recorded. The paw sensitivity threshold was regarded as the minimum force for leading to a strong and immediate withdrawal reflex of the paw. Motion-related random movements were not regarded as a withdrawal reaction. Stimulation was applied on each right posterior paw every 5 s. Tests were repeated 5 times and the final results were obtained by calculating the average value. PWT test was conducted to evaluate the hypersensitivity toward noxious mechanical stimulus. By detecting the pain threshold induced by mechanical stimulation, the smaller the value obtained, the more sensitive the rats are to pain, on the contrary, the larger the value obtained indicates that the treatment has a pain relief effect.

### Statistical analyses

All data was calculated as mean ± standard deviation (SD) from at least 3 independent experiments. The results were analyzed by Student’s *t* test or one-way analysis of variance (ANOVA) using the statistical software SPSS 19.0 (SPSS Inc., Chicago, IL). *p* < 0.05 was recognized as significantly different between groups. All histological images were analyzed by Image Pro Plus 6.0 (NIH, MD, USA).

## Results and discussion

### Hydrogel preparation

**Fig. 1:**
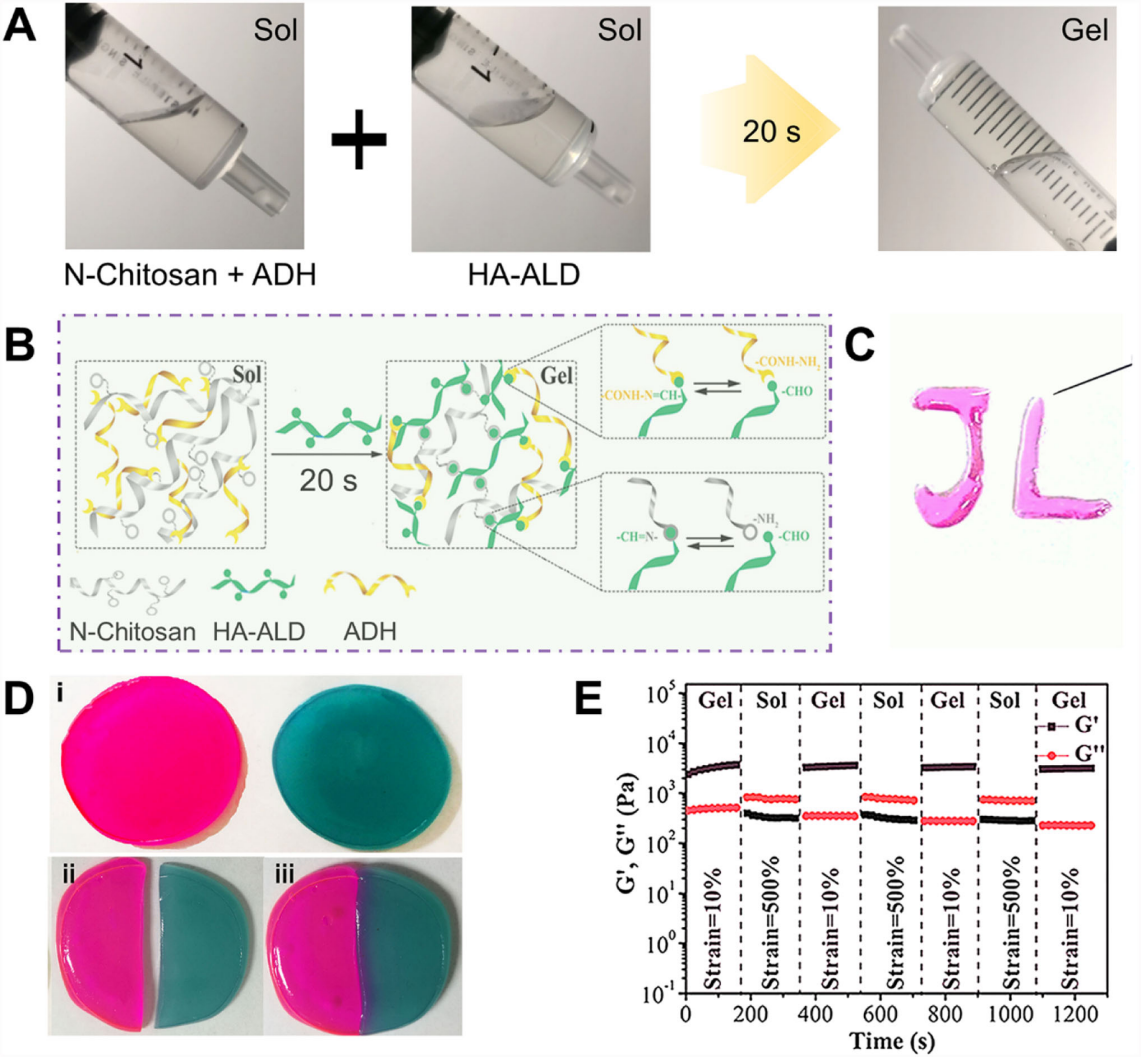
**A** The optical images of the gelation progress. The mixture of *N*-chitosan and ADH presented in the sol state at room temperature, and it experienced sol–gel transition after adding HA-ALD solution for about 20 s. The mixture solution transformed into the hydrogel without flow upon inversion. **B** The overall synthetic process of hydrogels. **C** The optical image of the injectable property. **D** The macroscopic self‐healing procedures of the hydrogel. **E** Rheological recovery tests of the hydrogel. Each strain interval was kept as 150 s. i, Complete morphology of hydrogels. ii, Hydrogel is cut by external force. iii, Self healing phenomenon occurs after hydrogel cutting by external force

To mimic essential characteristics of the native extracellular matrix (ECM), including water-rich property, dynamic modulation of 3D network structure, and concerted elasticity, we synthesized self-healing hydrogels for IA injection. Functional *N*-carboxyethyl chitosan was prepared via Michael’s reaction, and HA was chemically modified with aldehyde groups (HA-ALD). As shown in Fig. 1A, the mixture of *N*-chitosan and ADH presented with the sol state at room temperature, and it experienced sol–gel alteration after adding HA-ALD solution for about 20 s. Then, the mixture solution converted into the colorless transparent hydrogel, which would not flow upon inversion. The mechanism of sol‐to‐gel phase transformation process was ascribed to acylhydrazone (hydrazine groups of ADH reacted with HA-ALD) and imine bonds (amino groups of *N*-chitosan reacted with HA-ALD), respectively (Fig. 1B). Therefore, the hydrogel can be conducted easily by merging diluted solutions of *N*-chitosan, HA-ALD, and ADH (worked as a cross-linker) at the room temperature. Once the *N*-chitosan and ADH were mixed with HA‐ALD, the mixture could be extruded through a dual channel syringe with the 26‐gauge needle without blocking, and make steady hydrogels in just few seconds under the condition of room temperature (Fig. 1C).

The macroscopic flow and self-healing properties of hydrogels are demonstrated in Fig. 1D. The hydrogels were divided into two halves physically, and consequently the fresh halves were brought into connection. After 2 h of repair, the two separated hydrogels could form a complete hydrogel. The rheological recovery tests were conducted to quantitatively domenstrate the self-healing performance. The consecutive shear strain was changed between 500% and 10% continue for 200 s, separately. As displayed in Fig. 1E, when the shear strain was at 500%, shear thinning property of hydrogels was found that G″ was larger than G′. However, if the repeated shear strain was decreased to 10% (namely at lower shear strain), the G′ and G″ expeditiously almost reverted to their original values during 150 s time span. These results demonstrated that this novel hydrogel has autonomous self‐healing property when subject to oscillatory shear strain. This self-healing ability ensures the hydrogel once injected at the aimed sites, the broken hydrogel fragments have the ability to rescue their initial mechanical strength when the forces are decreased. This outstanding self-healing property was promised by the reversible dynamic bonds, since the dangling acylhydrazone and imine groups reacted to reform a single and intact gel during the healing process [15, 16].

This injectable property ensures the feasibility of IA injection in OA condition. The kind of soft materials have essential self-healing quality and can sustain their functions and structures even with external impair. The characteristic is favorable and important in maintaining the integrity of network structures and mechanical properties of bulk gels containing active biological drugs/cells and relieving them to target sites [19, 20]. Moreover, conventional injectable hydrogels are prone to decrease or even lose their mechanical strength after injection, which then cause delivery failure [21, 22]. However, this kind of self-healing hydrogel can flow through a needle under force and retain steady condition after injection, which are extremely desirable for biological applications of IA injection.

### Biocompatibility and degradation of the hydrogel

**Fig. 2:**
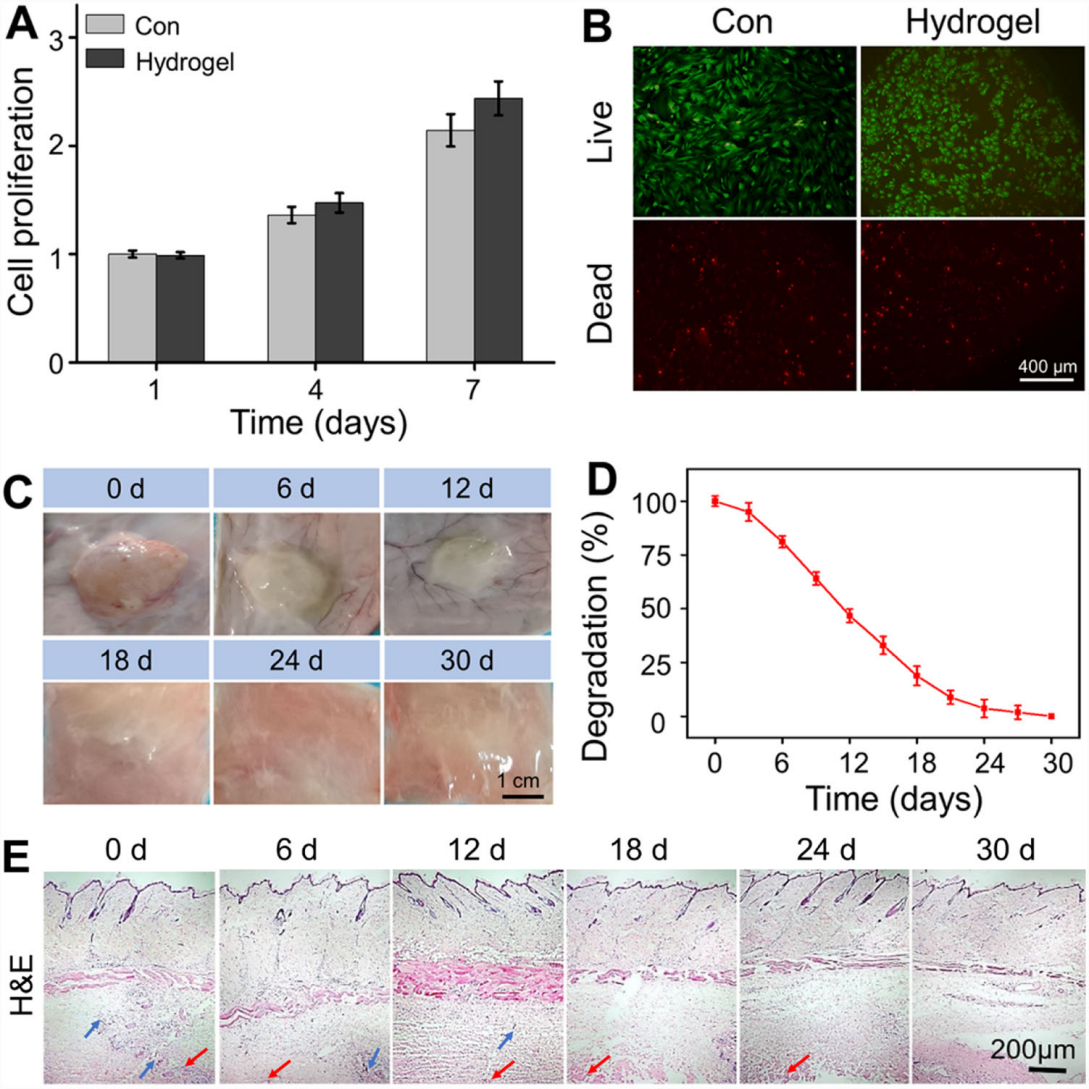
**A** Chondrocytes proliferation in the plate (Con) and hydrogel at 1, 4, and 7 d. **B** Calcein AM/PI staining of live cells (green) and dead cells (red). **C** General images of hydrogel degradation after subcutaneous injection for 0, 6, 12, 18, 24, and 30 days. **D** Quantitative statistics of undegraded hydrogel weight. **E** H&E staining showed no obvious inflammatory reaction in the surrounding muscles and subcutaneous tissues after subcutaneous injection of hydrogel. Blue arrows indicated the inflammatory cells and red arrows represented the hydrogel fragments

In order to assess the cell proliferation and viability, CCK-8 analysis and Calcein AM/PI staining were carried out. As shown in Fig. 2A, there was no significant difference in the number of cells on the plate (Con group) and on the surface of prepared hydrogel after 1 and 4 days of culture. While incubating for 14 days, the proliferation of chondrocytes on the hydrogel indicated an increasing trend compared with the Con group. The fluorescence microscope images clearly evidenced that the chondrocytes could maintain good cell viability on the hydrogel (Fig. 2B). These results revealed that the chitosan-based hydrogel had good biocompatibility, and was without cytotoxicity to chondrocytes. It has been acknowledged that hydrogel is similar to natural ECM which is conducive to cell growth. Furthermore, the biomimetic 3D microenvironment of hydrogels facilitates the efficient transport of nutrients and oxygen, promoting cell proliferation and recruitment [23]. The *in vivo* degradation was performed on SD rats. As shown in Fig. 2C, D, it took about 24–30 days from subcutaneous injecting hydrogel to complete degradation. The host response to the hydrogel is another crucial point for the implanted materials, and *in vivo* biocompatibility of hydrogel was detected by H&E staining. Because the undegraded hydrogel was stripped and weighed to calculate the degradation rate, only a small amount of dispersed hydrogel fragments (red arrows) could be seen in the dermis. A very slight inflammation was detected 20 min after subcutaneous injection (recorded as 0 day), based on a small amount of inflammatory cell infiltration (blue arrow). It may be that the hydrogel acts as a foreign body, causing a certain host immune response. The inflammation was gradually alleviated along with the hydrogel degradation. After 12 days, there was no obviously inflammatory cells infiltration at the injection site and surrounding tissue (Fig. 2E), indicated that the hydrogel has good biocompatibility. Generally, these results suggested that the drug delivery system possessed good biocompatibility and non-cytotoxicity *in vitro* and *in vivo*.

### Induction of osteoarthritis and experimental design

**Fig. 3:**
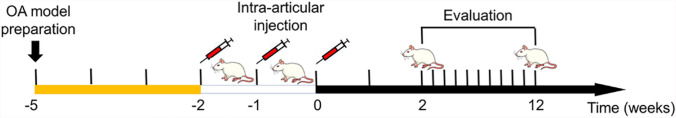
Timeline of *in vivo* experimental protocol

Unilateral OA rat models were induced by single monoiodoacetate (MIA) injection. Three weeks after MIA induction, treatment started and IA injection of saline, HA, and hydrogel were administered in the right knee once a week for three times. After 2 weeks (short-term effect) and 12 weeks (long-term effect) of IA treatment, the concentration of inflammatory factors in the joint fluid and fluorescence staining of the cartilage were observed to detect the inflammatory microenvironment of joints. Subsequently, pain behavior studies and cartilage tissue sections were performed. Detailed timeline of animal experimental design is displayed in Fig. 3.

### IA injection of hydrogel reduces inflammation

Proinflammatory cytokines are seemed as fundamental mediators in the initiation and development of OA. TNF-α, IL-1β, IL-6 and IL-17 are among the essential cytokines enrolled in the pathophysiology of OA [3, 24]. To study the inflammatory reactions in OA microenvironment after IA injection, these cytokines expression in synovial fluid and cartilage were analyzed quantitatively.

**Fig. 4:**
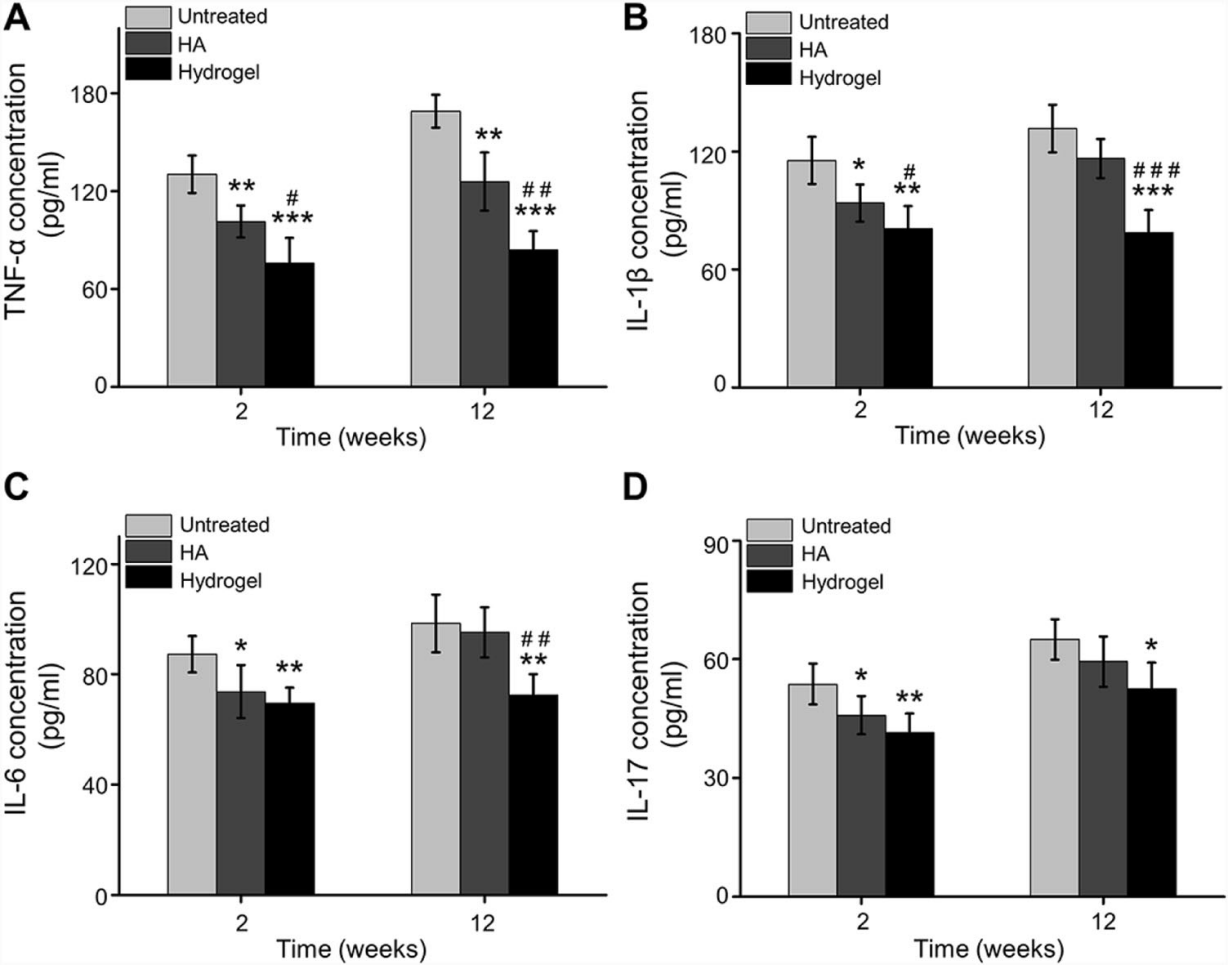
Inflammatory cytokines expressed in synovial fluid. **A** TNF-α, **B** IL-1β, **C** IL-6, **D** IL-17 concentration in synovial fluid at 2 weeks and 12 weeks post-injection (*p < 0.05, **p < 0.01, ***p < 0.001 compared with the Untreated group; ^#^*p* < 0.05, ^##^*p* < 0.01, ^###^*p* < 0.001 compared with the HA group)

TNF-α, as a critical cytokine related to OA, plays an essential role in facilitating synovial cells proliferation, enhancing other pro-inflammatory cytokines expression [25]. TNF-α can induce the expression of inflammatory cytokines, such as IL-1β and IL-6, and promote leukocyte migration. In addition, TNF-α can activate the activity of neutrophils and eosinophils, and induce synovial cells and/or chondrocytes to produce tissue degrading enzymes [26, 27]. Previous studies have shown that excessive expression of TNF-α can induce cascade inflammatory reactions, thus causing pain and cartilage destruction [28]. In this study, expression levels of TNF-α in synovial fluid were significantly inhibited in IA with chitosan-based hydrogel compared with animals treated with HA or Untreated groups at 2 weeks and 12 weeks. Simultaneously, the expression of TNF-α in the OA synovial fluid injected with HA was also lower than Untreated group at 2 weeks and 12 weeks (Fig. 4A). In addition, IL-1β, IL-6, and IL-17 are also detected to evaluate the inflammatory condition of OA. IL-1β is significantly upregulated in OA, inducing cartilage catabolism and decreasing its anabolism, enhancing expression of chemokines and promoting the apoptosis of chondrocytes. Furthermore, IL-6 plays a role in the inflammation and degeneration of the knee joint, and causes disorder in subchondral bone metabolism and inhibits the expression of collagen [29]. IL-17 was tightly related to cartilage defects and bone marrow lesions in patients suffering from an enhanced inflammatory status [30]. Herein, for HA injection, the expression of IL-1β (Fig. 4B), IL-6 (Fig. 4C), and IL-17 (Fig. 4D) in synovial fluid was significantly inhibited at 2 weeks post-injection, but this inhibition effect was with no significant difference in long-term observation (12 weeks post-injection) compared with the Untreated group. However, the levels of IL-1β (Fig. 4B), IL-6 (Fig. 4C), and IL-17 (Fig. 4D) in synovial fluid were significantly inhibited when treated with hydrogel compared with Untreated group at 2 weeks and 12 weeks. Simultaneously, compared with HA group, the down-regulated expression levels of IL-1β and IL-6 were also observed in synovial fluid in the hydrogel group at 12 weeks post-injection.

**Fig. 5:**
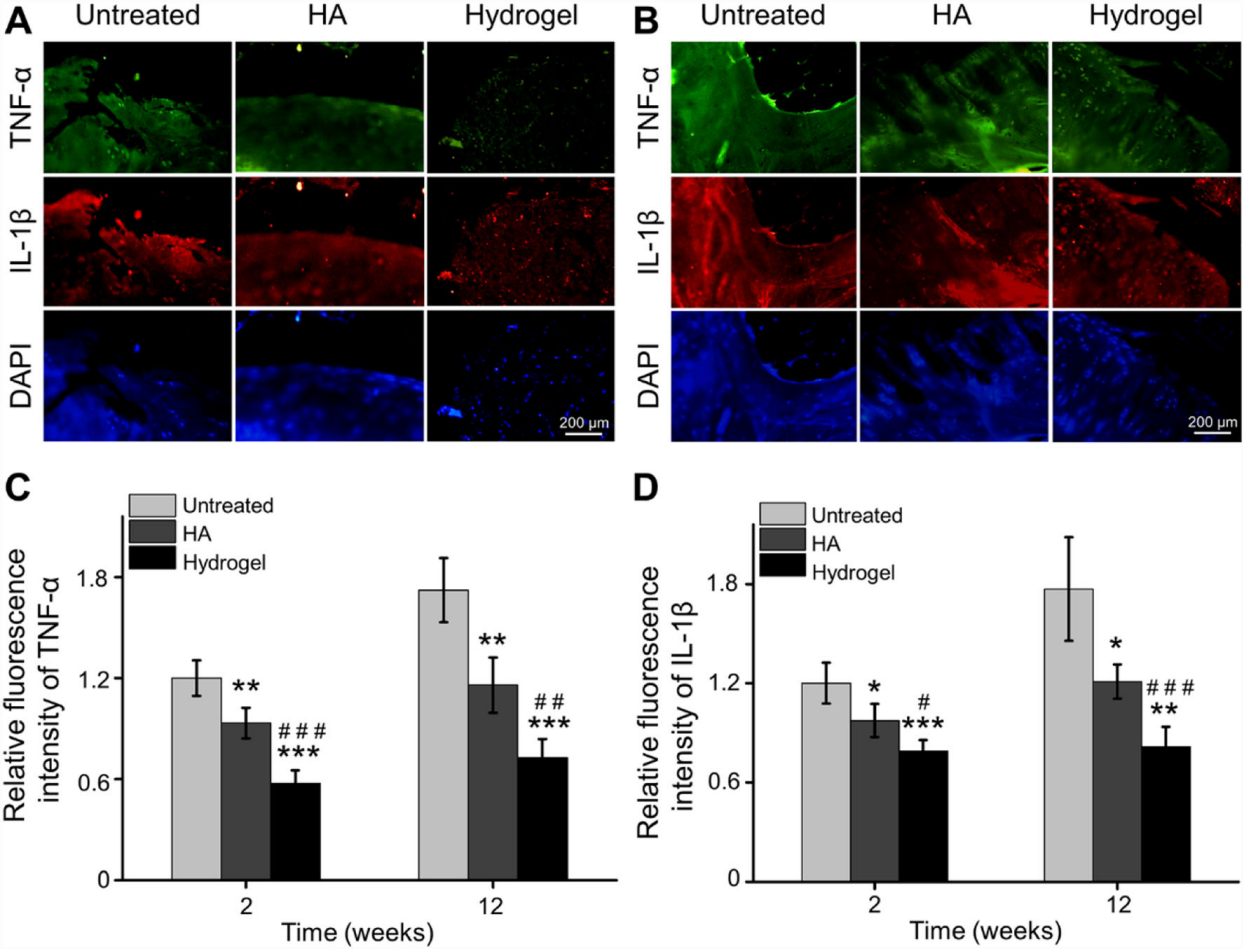
Inhibiting inflammatory cytokines expression in cartilage. **A** Representative immunofluorescence images of TNF-α and IL-1β expression in cartilage 2 weeks post-injection. **B** Representative immunofluorescence images of TNF-α and IL-1β expression in cartilage 12 weeks post-injection. **C** Quantitative statistics of TNF-α expression in cartilage 2 weeks and 12 weeks post-injection. **D** Quantitative statistics of IL-1β expression in cartilage 2 weeks and 12 weeks post-injection (*p < 0.05, **p < 0.01, ***p < 0.001 compared with the Untreated group; ^#^*p* < 0.05, ^##^*p* < 0.01, ^###^*p* < 0.001 compared with the HA group)

**Fig. 6:**
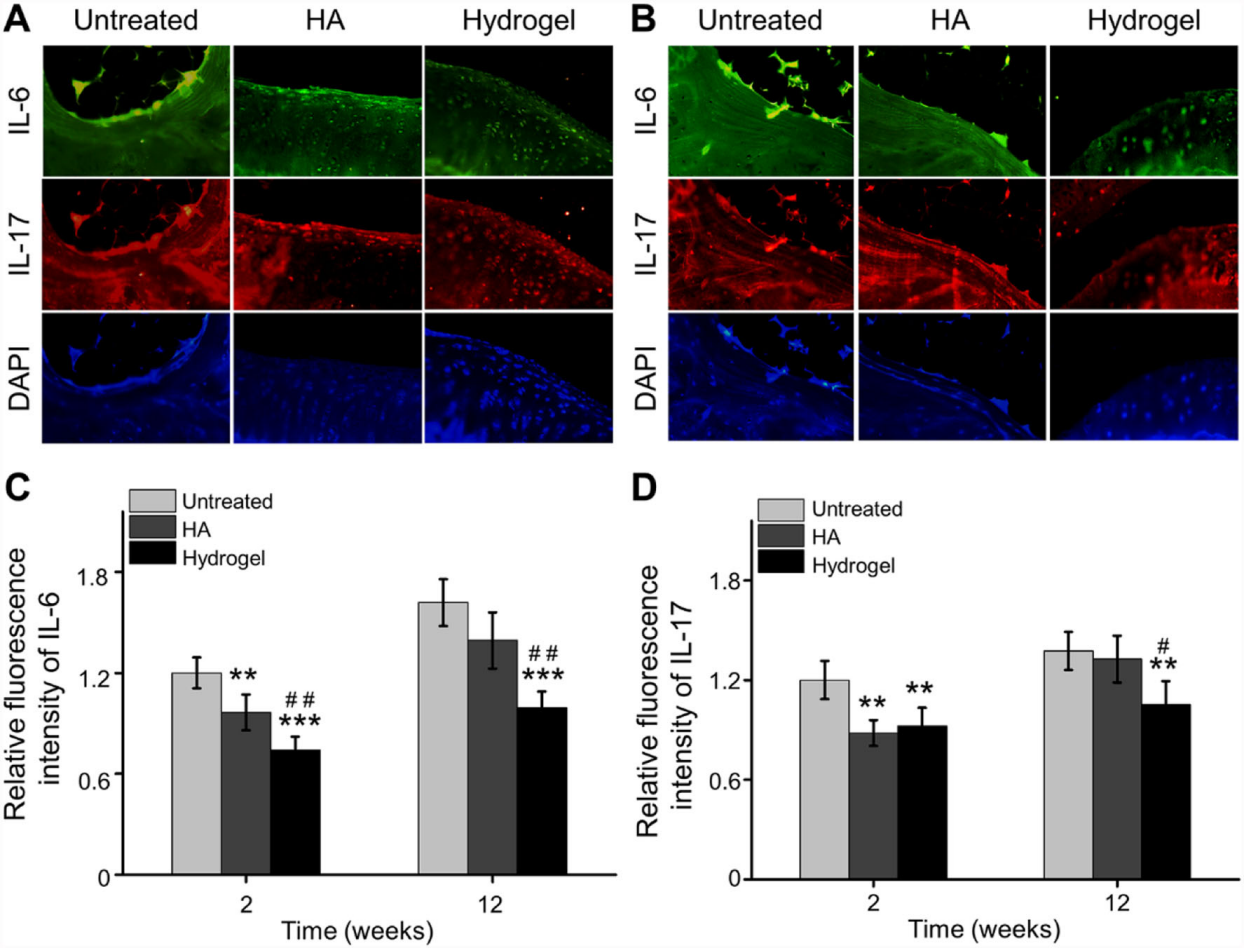
Inhibited inflammatory cytokines expression in cartilage. **A** Representative immunofluorescence images of IL-6 and IL-17 expression in cartilage at 2 weeks post-injection. **B** Representative immunofluorescence images of IL-6 and IL-17 expression in cartilage at 12 weeks post-injection. **C** Quantitative analysis of IL-6 expression in cartilage at 2 weeks and 12 weeks post-injection. **D** Quantitative calculation of IL-17 expression in cartilage at 2 weeks and 12 weeks post-injection (**p < 0.01, ***p < 0.001 compared with the Untreated group; ^#^*p* < 0.05, ^##^*p* < 0.01 compared with the HA group)

In order to further analyze the expression of inflammatory cytokines in cartilage, immunofluorescence staining of TNF-α, IL-1β, IL-6 and IL-17 was performed at 6 weeks and 12 weeks post-injection. Based on fluorescence images and quantitative analysis by IPP, the relative fluorescence intensity of TNF-α and IL-1β in the HA group was decreased at 2 and 12 weeks after injection (Fig. 5A–D), but the inhibited effect of IL-6 and IL-17 could not sustain until the 12th week, compared with the Untreated group (Fig. 6A–D). For the OAwXT rats treated with chitosan-based hydrogel, this anti-inflammatory effect kept for up to 12 weeks compared with Untreated group and HA group (Figs. 5, 6).

It has been reported an increase in inflammatory cytokines levels, including TNF-α, IL-1β, IL-6, and IL-17, associated with the development of OA in rats [31, 32]. In addition to recovering the elasticity of synovial fluid, HA has a chondroprotective effect and can regulate joint inflammation and pain responses [33]. However, this improvement is not sustaining due to the rapid degradation and single action of HA. Chitosan is considered as an ideal material for hydrogels for its biocompatibility, progressive degradability, non-toxic, biological activity, anti-inflammatory effects and antibacterial activity. A chitosan ehyaluronate hybrid gel was recently identified to postpone the development of OA model [34]. Furthermore, *in vitro* studies have demonstrated that chitosan enhanced the expression of cartilage matrix components secreted by chondrocytes [35, 36] and suppressed the production of inflammatory and catabolic mediators [37]. Herein, this chitosan-based hydrogel prepared by crosslinking of *N*-chitosan and ADH with HA-ALD processed an enhanced and lasting anti-inflammatory effect, thus providing a suitable microenvironment for reducing cartilage destruction [10].

### IA injection of hydrogel alleviates cartilage degeneration

In patients with OA, articular cartilage destruction and disability caused by joint dysfunction are the most serious consequences. Therefore, for the treatment of OA, it is essential to protect and retard the articular cartilage. Among conventional OA treatments, IA viscosupplementation with HA is used to rescue joint viscoelasticity. While, the rapid elimination and clearance of HA may restrict its application. In order to improve the efficacy of HA within the joints, chitosan as a potentially chondroprotective additive incorporating into the therapeutic agents for IA can provide a significant improvement in knee articular cartilage degeneration and synovium inflammation in OA rat model [38].

**Fig. 7:**
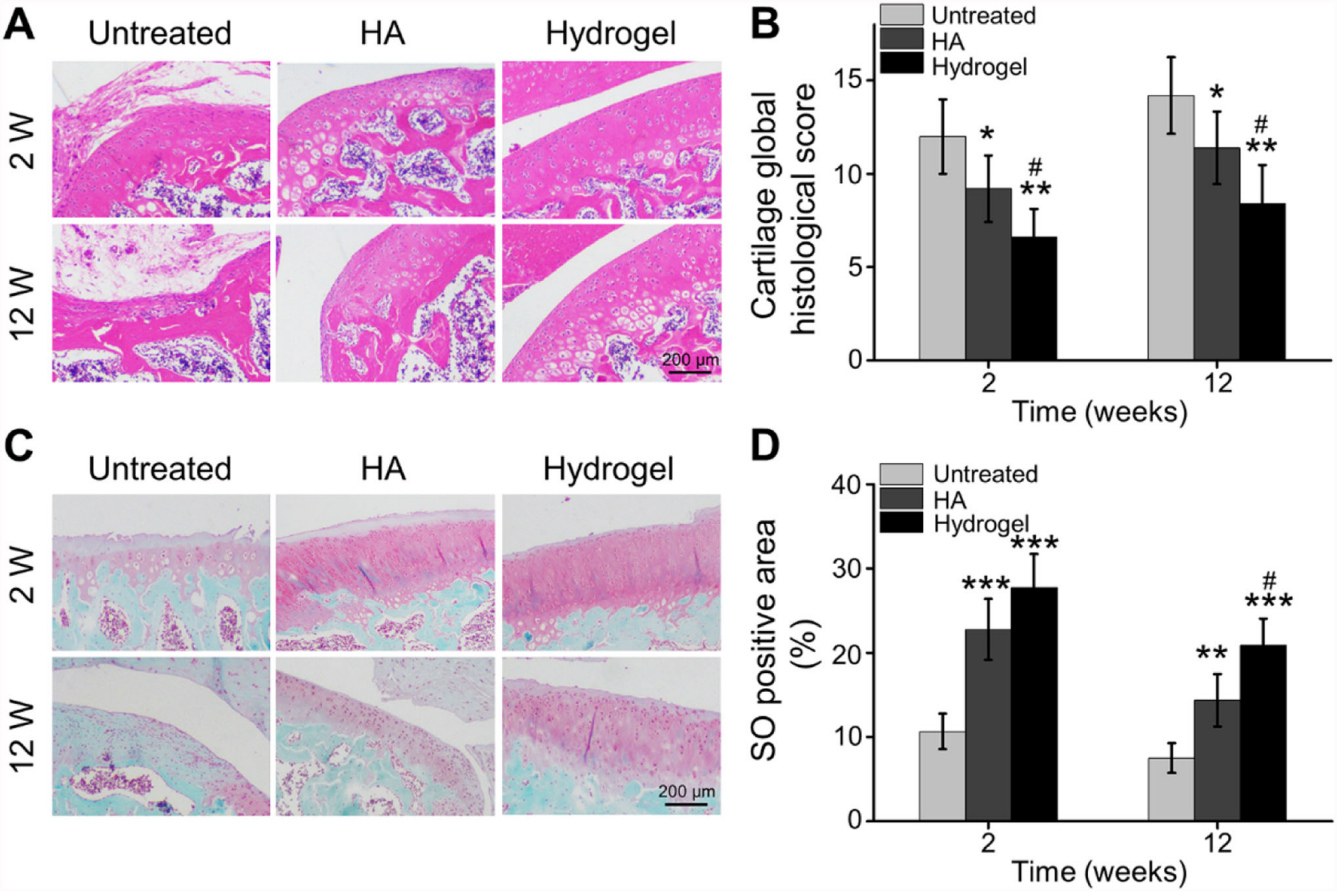
Histological analyses of articular cartilage. **A** H&E staining of articular cartilage at 2 and 12 weeks after IA injection. **B** Quantitative analysis of Safranin-O positive area in the articular surface. **C** Safranin O staining of articular cartilage at 2 and 12 weeks after IA injection. **D** Global quantitative histological score of different treatments (*p < 0.01, **p < 0.01, ***p < 0.001 compared with the Untreated group; ^#^*p* < 0.05 compared with the HA group)

As shown in Fig. 7A, the H&E staining indicated that the articular surface was unsmooth, even some cartilages were missing and damaged, marked fibrosis, and the surface cells of cartilage were necrosis in the Untreated group, especially at 12 weeks. In the HA group, the cartilage surface was relatively intact at 2 weeks, but the cells were arranged loosely and irregularly, and suspected fibrous components could be seen on the surface of some cartilages at 12 weeks. Hydrogel-treated rats showed smooth and intact surface with thick cartilage layer, and the cells were arranged regularly. Quantitative scoring demonstrated a significant upregulation in the histological score following MIA-induced OA over time. On the other hand, both HA treatment and hydrogel injection caused a significant decrease of score compared to Untreated group whether 2 weeks or 12 weeks after IA injection. Importantly, hydrogel group significantly improved of histological scores compared to the HA treatment at 2 weeks and 12 weeks post-injection (Fig. 7B).

Glycosaminoglycans (GAGs) are crucial constituents of cartilage, working as the synovial fluid lubricant in joints. The articular knee joint of intra-articularly injected OA rats was stained with Safranin-O to assess potential therapeutic influence on the cartilage (Fig. 7C). After Safranin-O staining, GAG deposits turned red in the articular knee joint [39]. The loss of cartilage rich in polysaccharide was significant in the Untreated group. The degeneration of cartilage was also obvious in HA group after 12 weeks. However, the hydrogel injection group played a good role in preventing cartilage loss. Quantitative analysis of Safranin-O positive area indicated that GAG-positive magnitude in Untreated, HA, and hydrogel groups were 10.65 ± 2.12%, 22.76 ± 3.60%, and 27.72 ± 3.97% after 2 weeks post-injection, and 7.49 ± 1.78%, 14.36 ± 3.12%, and 20.88 ± 3.15% after 12 weeks post-injection, respectively (Fig. 7D). These results indicated that OA rats treated with IA injection of hydrogel significantly retarded cartilage degeneration, compared to the Untreated group and HA group.

### IA injection of hydrogel relieves pain

**Fig. 8:**
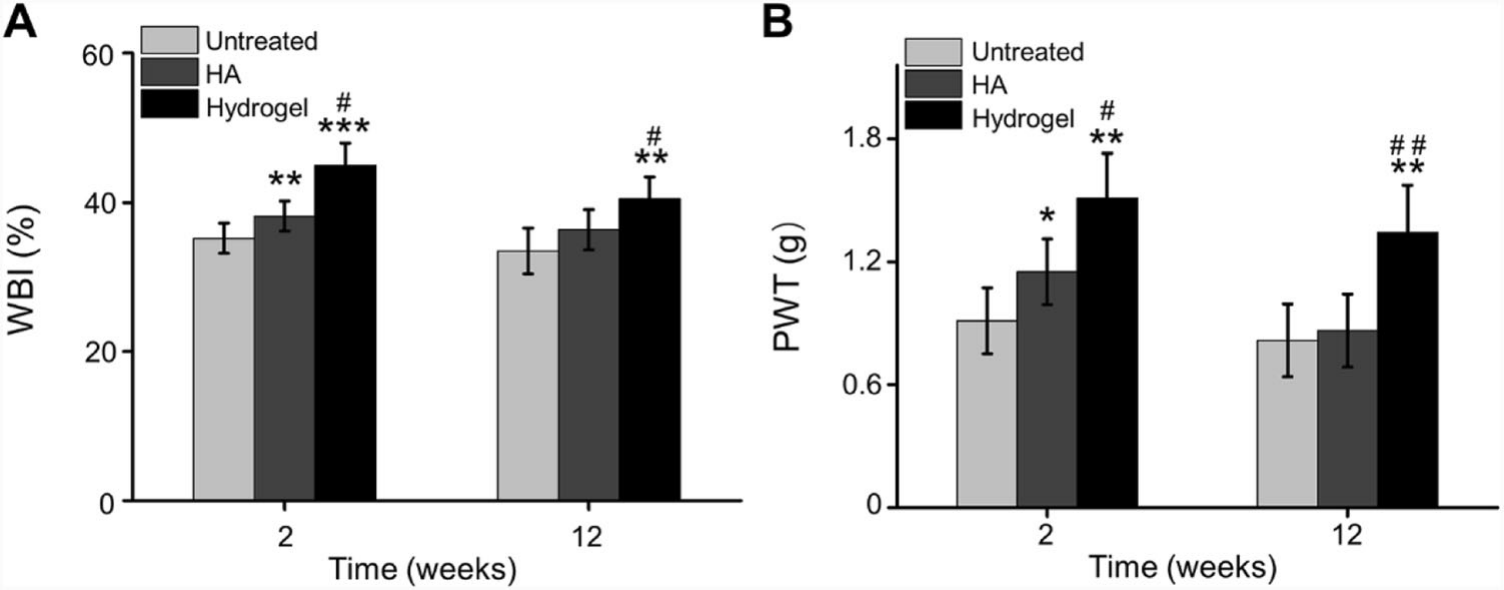
Pain-related behavior after IA injection. **A** Weight-bearing capacity was calculated by weight-bearing index (WBI). **B** PWT was measured to test mechanical allodynia (*p < 0.01, **p < 0.01, ***p < 0.001 compared with the Untreated group; ^#^*p* < 0.05, ^##^*p* < 0.01 compared with the HA group)

The weight-bearing index (WBI) was observed at 2 and 12 weeks after IA injection of 0.4 mL therapeutic agents into the OA knees. Quantitatively, the WBI value of the Untreated, HA, and hydrogel groups were 35.21 ± 2.03%, 38.19 ± 2.01%, and 45.03 ± 2.88% at 2 weeks, and 33.49 ± 3.08%, 36.36 ± 2.70%, and 40.49 ± 2.94% at 12 weeks, respectively. HA intra-articular injection into joints can relieve pain by inhibiting the inflammatory response with the direct action of HA or through reinforcing the viscosity of joint fluid [40]. Therefore, the WBI value of HA group was significantly higher than Untreated group at 2 weeks (*p* < 0.01). Due to the incorporation of chitosan, the injection formulation was optimized, and the novel prepared hydrogel has good mechanical properties and self-healing properties benefiting to pain relief. At both 2- and 12-weeks post-injection, the weight bearing capacity of hydrogel group showed a better result compared with Untreated and HA groups (Fig. 8A).

In addition, the ipsilateral paw withdrawal threshold (PWT) of the hydrogel group was 12.60 ± 1.82 g on week 2 and 11.2 ± 1.92 g on week 12, respectively, which were significant higher compared to the Untreated and HA groups (*p* < 0.05). While the PWT of HA group was only higher at the second week (*p* < 0.05), and there was no difference at 12 weeks after injection, compared to the Untreated group (Fig. 8B).

Peripheral pain mechanisms involve the direct activation of nociceptors, and sensitization of nociceptors induced by the inflammation in articular cavity [41, 42]. Local immune cells relieve inflammatory cytokines and additional molecular mediators that work on the peripheral nerve terminals of nociceptor neurons [43]. Responding to the inflammatory mediators, intracellular signaling pathways cause a phosphorylation cascade, which decreases the threshold for nociceptor neurons to activate action potentials, eventually resulting in heightened pain sensitivity [44]. The inflammatory cytokines, such as TNF-α, IL-1β, IL-6, and IL-17, can directly change the responses of nociceptive neurons [45]. Herein, pain relief after IA injection may be attributed to sustained anti-inflammatory effects of this chitosan-based hydrogel.

In conclusion, an injectable and self-healing hydrogel was successfully produced by in situ crosslinking of *N*-chitosan and ADH with HA-ALD for IA injection therapy in OA. Compared with the traditional treatment of IA injection of HA, this chitosan-based supramolecular hydrogel provided with durable curative effect of regulating inflammatory microenvironment, retarding cartilage destruction, and relieving pain.
